# Redox Status and Bioenergetics Liaison in Cancer and Neurodegeneration

**DOI:** 10.1155/2012/659645

**Published:** 2012-09-11

**Authors:** Giuseppe Filomeni, Juan P. Bolaños, Pier Giorgio Mastroberardino

**Affiliations:** ^1^Department of Biology, University of Rome “Tor Vergata”, Via della Ricerca Scientifica, 00133 Rome, Italy; ^2^Research Centre IRCCS San Raffaele Pisana, Via di Val Cannuta 247, 00166 Rome, Italy; ^3^Department of Biochemistry and Molecular Biology, Institute of Functional Biology and Genomics (IBFG), IBSAL, CSIC, University of Salamanca, Calle Zacarias Gonzalez 2, 37007 Salamanca, Spain; ^4^Department of Genetics, Erasmus Medical Center, Dr. Molewaterplein 50, 3015 GE Rotterdam, The Netherlands

During the last decades, the involvement of redox reactions in each aspect of cellular physiology has emerged, rapidly steadied, and is currently assuming extensive connotations. From a mere pathological condition leading to widespread biomolecules damage and cell degeneration, (over)production of reactive oxygen and nitrogen species (ROS and RNS, resp.) is now also deemed to be among the early upstream events of signal transduction pathways governing cellular response. It is now well established that the thiol moiety of reactive cysteines is the main molecular switch selected by the evolution to transduce a redox signal. Even at physiological pH, the sulfhydryl group of these residues is present under thiolate form and primed to be modified by ROS and RNS in a reversible manner. *S*-nitrosylation, *S*-hydroxylation, and *S*-glutathionylation are, indeed, all oxidations that meet the conditions of specificity and reversibility required for a chemical modification (signal) being transduced in a biological event (response).

In this special issue, the double-faced role of nitroxidative stress (deleterious, or signal mediator) has been carefully considered, mostly in regards to the implications that it could have in neurodegeneration. The paper by R. P. Guttmann and T. J. Powell underlines this aspect elucidating, in the first part, the biochemical basis of the high susceptibility of brain towards redox stress and providing, in the second one, a very clear overview of the possible sources, mechanisms, and targets of oxidative stress, namely cysteine-containing enzymes, which can be implicated in the onset of neurodegenerative diseases. Although it is still unclear whether oxidative stress is the primary initiating event or a secondary effect related to other pathological conditions, its role in neurodegenerative diseases is confirmed by the evidence that the brain is particularly prone to ROS and RNS production and susceptible to their harmful effects. Its high metabolic rate and content of oxidizable molecules, (e.g., neurotransmitters), as well as the high concentration of redox-active metals (e.g., iron and copper) and the limiting levels of antioxidants, mainly glutathione (GSH), are features concurring to exacerbate oxidative challenges in the central nervous system (CNS). These aspects have been fully covered in a series of papers included in this special issue. In particular, R. B. Mounsey and P. Teismann dissect out in depth the contribution of iron accumulation in Parkinson's disease (PD) and debate the promising on-going pharmacological approaches aimed at reversing this phenomenon, including the use of both synthetic and natural iron chelators. C. Consales et al. deal with the emerging lines of evidence linking electromagnetic fields (EMF) and neurodegenerative diseases onset. They discuss about the still not reconciled results reporting both neuroprotective and pro-oxidant role of EMF and critically assess methodological limitations that could affect pathophysiological relevance of any alteration found in EMF-exposed biological systems. The paper by W. Li et al. complements the information on the role of environmental factors in exacerbating pro-oxidant conditions in neurodegeneration, by providing a very interesting overview of the role of GSH in brain homeostasis and protection. Remarkably, besides the role of GSH in driving cell signaling and redox state maintenance, the authors shed new light on its involvement in the control of neurovascular function. GSH-mediated protection against endothelial cell injury and promotion of postdamage cell proliferation in endothelial repair are discussed on the basis of the very recent lines of evidence reporting the involvement of GSH-dependent redox modification (e.g., *S*-glutathionylation) in cerebral microvascular biology and pathobiology, as well as in oxidative stress-associated neurovascular disorders, such as stroke and diabetes. The paper by L. Rossi et al. concludes this part of the special issue revisiting the current opinion regarding the role of oxidative stress in motor neuron diseases (MNDs). Interestingly the authors provide convincing evidence that increased oxidative stress, mitochondrial damage, and altered gene expression (due to epigenetic defects and RNA dysmetabolism) are common features of different MDNs that could underlie MDNs pathogenesis. 

Amongst the environmental factors contributing to neurodegenerative diseases, chemicals and drugs affecting the mitochondrial electron transport chain (e.g., paraquat and rotenone) are frequently reported to induce apoptotic cell death. However, endogenous molecules have also been demonstrated to be detrimental if produced in excess, such as glutamate or certain cytokines. Recently, the macrophage migration inhibitory factor (MIF), a macrophage-produced cytokine, has been reported to be involved in disease pathogeneses. In this special issue, N. E. Savaskan provide an overview of our current knowledge about MIF action, especially regarding its potential role in brain disease and redox regulation in apoptosis. Neuronal apoptosis is an important mechanism contributing to the selective loss of cell populations in specific regions of the CNS. It can take place *via* the mitochondrial pathway, the main step of which is the release of the mitochondrial respiration mediator cytochrome *c* within the cytosol. On this topic, L. Berghella and E. Ferraro provide an original research paper dissecting out in depth the sequence of events of neuronal apoptosis. They provide evidence that cytosolic cytochrome *c* has no role in chromatin condensation and that it is not a primary cause of mitochondrial respiration impairment, which occurs long before its release. These results are in agreement with the current knowledge suggesting that the effects of apoptotic-associated dysfunction on mitochondrial respiration are not mere epiphenomena, but are rather crucial for proper execution of the apoptotic program. In particular, the massive production of ROS and the generation of pro-oxidant state favoring the activation of downstream apoptotic factors are gaining increasing importance. Indeed, mitochondria represent an important endogenous source of ROS that are physiologically generated at the mitochondrial electron transport chain. The sustained and long-lasting production of ROS is the principal cause of nitroxidative damage to biomolecules. It can produce DNA damage and, in the long term, it can give rise to established somatic mutations. These effects can contribute to neoplastic transformation and are detrimental to neuronal viability. In this special issue, D. De Zio et al. deeply address this topic, providing a detailed overview regarding the occurrence of oxidative DNA damage in neurons and how it is specifically counteracted by the activity of DNA repair systems. The authors discuss how the unbalance between these processes results in neurodevelopment disorders and neurodegenerative diseases and focus on the role of the nuclear factor Ku70/80 as pivotal mediator of neuronal viability. Likewise, S. Gonfloni et al. discuss the role of the c-Abl tyrosine kinase, as its increased activation has been reported in several neurological disorders. They provide a paper in which they propose that c-Abl signaling contributes to modulate molecular events at the interface between oxidative stress, metabolism, and DNA damage, suggesting possible therapeutic strategies targeting c-Abl for the treatment of neurodegenerative diseases.

Besides being an important site for ROS production, the mitochondrial electron transport chain generates the proton motive force that is used for ATP synthesis by the *F*_0_/*F*_1_ ATP synthase. The mitochondrial transmembrane potential (ΔΨ_*m*_) is a measure of the proton electrochemical gradient generated across the mitochondrial inner membrane and represents a good indicator of the energy status of the mitochondrion. Under certain pathophysiological conditions, such as hypoxia-ischemia, ΔΨ_*m*_ can be transiently preserved at the expense of ATP. This is brought about by the reverse reaction of *F*_0_/*F*_1_ ATPase, which can hydrolyze glycolytically generated ATP to pump protons into the intermembrane space, thus maintaining the ΔΨ_*m*_. In this special issue, D. Faccenda and M. Campanella provide an up-to-date overview of mitochondrial energetics and the molecular characterization of *F*_0_/*F*_1_ ATPase, highlighting its function and the regulatory events that trigger either the ATP-synthesizing or the ATP-hydrolyzing activities. The authors focus on the role of the inhibitory factor 1 (IF_1_), a polypeptide that responds to mitochondrial intermembrane space acidification by inhibiting the ATP-hydrolyzing activity of the *F*_0_/*F*_1_ ATPase; this occurs by stabilizing its dimerization status *via* a molecular link between two *F*_1_ domains. This molecular rearrangement remodels the mitochondrial cristae structure and, consequently, regulates organelle morphology, thus involving IF_1_ in neurodegeneration and cancer. Given that mitochondrial healthy state strongly impacts on the generation of intracellular nitroxidative stress conditions, chronic alterations of mitochondrial homeostasis are among the events concurring to the onset of several pathological states, namely cancer and neurodegenerative diseases. Nevertheless, it should be also reminded that mitochondria have evolved specific molecular quality control mechanisms to counteract the damaging effects of nitroxidative stress. In this special issue E. Desideri and L. M. Martins provide an overview of the current knowledge of mitochondrial quality control and discuss the pivotal role that PTEN-induced putative kinase 1 (PINK1) and the protease high-temperature-regulated A2 (HTRA2) might play in this context. In particular, they report evidence and speculate about the possibility that defects in their activity might contribute to PD onset.

From a metabolic perspective, neurons and cancer cells exhibit sharp differences. Neurons strictly depend on efficient OXPHOS for ATP production; here, mitochondrial respiration does not depend directly on glucose availability and is rather fueled by pyruvate derived from glia-provided lactate in a Cori's cycle-like manner. Glucose taken up by neurons is instead mainly redirected to the pentose phosphate pathway, allowing the generation of NADPH to sustain the indispensable sulfhydryl reductive pathways and antioxidant response. By contrast, tumor cells synthesize ATP almost entirely by means of glycolysis even in normoxic conditions, when oxygen availability is not restricted (the “Warburg effect”). This condition represents a major change of the entire metabolic reprogramming typical of tumors and allows cancer cells to grow even under low oxygen tension, when tumor vascularization is still incomplete, and therefore, local vessels fail to supply adequate amount of oxygen. Upregulation of the glycolytic pathway is therefore a selective advantage to sustain the ATP demand required for tumor proliferation under hypoxic conditions. M. Fernandes de Oliveira et al. comprehensively review the mitochondrial biochemical pathways underlying bioenergetics, ATP production, and ROS generation in cancer. The authors also discuss the connection between mitochondrial dynamics and function, and how this is related to apoptotic cell response. In detail, they give emphasis to the role of ROS and RNS in the activation of redox-sensitive transcription factors and protein kinases in tumor cells, and how they can be manipulated to selectively kill them. Along this line, M. Soga et al. discuss recent data underlying the tight liaison between redox state alteration and mitogen-activated protein kinases (MAPKs) activation, namely the apoptosis signal-regulating kinase 1 (ASK1). Being the Ichijo's laboratory among the pioneers to have characterized the role of thioredoxin (Trx) as the regulatory partner of ASK1, in this paper, the authors revisit the main steps of this regulation and provide the new lines of evidence arguing for the involvement of ASK1 in several pathological states.

As previously mentioned, metabolic reprogramming of tumor cells synergizes with nitroxidative stress to maintain and develop tumor aggressiveness. T. Fiaschi and P. Chiarugi deepen this concept in their paper and extend it to the entire tumor microenvironment that, together with stromal cells, enhances pro-oxidant conditions and concurs to the establishment of a more aggressive and chemoinsensitive neoplastic phenotype. To corroborate the intrinsic connection between metabolism and tumorigenesis, S. Cardaci and M. R. Ciriolo discuss the causative role of mutations in three enzymes belonging to the tricarboxylic acid (TCA) cycle, succinate dehydrogenase (SDH), fumarate hydratase (FH), and isocitrate dehydrogenase (IDH). Together with K. Smolková and P. Ježek, who reviewed in detail the oncogenic potential of the mitochondrial isoform of IDH, these authors highlight how point mutations of these enzymes generate an hypoxia-like phenotype, affect cellular redox state, and interfere with DNA methylation dynamics, thereby revealing the main tumor-promoting effects of TCA cycle alterations. To complete this section, N. Berndt et al. develop a kinetic model aimed at predicting and quantifying mitochondrial OXPHOS efficiency and ROS production induced by the reduced activity of *α*-ketoglutarate dehydrogenase (KGDH). This condition occurs in several neurodegenerative diseases, such as PD and Alzheimer's disease (AD), and the results shown in this paper give strength to the hypothesis that decreased KGDH activity plays a pivotal role in the energetic failure of neuronal cells during neurodevelopment and neurodegeneration. KGDH is a multienzymatic complex responsible for the conversion of *α*-ketoglutarate into succinyl-CoA, a critical step in the TCA cycle. The TCA cycle is a central hub in metabolism; its functions are not restricted to energy conservation and involve afferent as well as efferent anabolic pathways. *α*-ketoglutarate is an important intermediate for afferent processes because it is the catabolic product of several amino acids and is also generated by deamination or transamination of glutamate. *α*-ketoglutarate is therefore essential for energy production from amino acid substrates. On the other hand, efferent pathways generate new amino acids from the sugars entering the TCA cycle, which are indispensable for cancer cells to synthesize new proteins and produce biomass. The levels of amino acids in the extracellular milieu are sensed by the multimolecular mTOR complex 1 (mTORC1) and transduced *via* well-known signaling axes that lead to normal cell growth or autophagy. Here, mTOR serves as master metabolic switch; when mTOR is active, it promotes anabolic metabolism repressing autophagy, and when it is inactive (e.g., during nutrient starvation) or inhibited (e.g., by rapamycin), cells switch to a catabolic mode. In the latter case, autophagy is activated to recycle proteins, whereas organelles and protein synthesis are repressed. Although autophagy is a homeostatic process for normal cells, it is also exploited by cancer cells to grow and survive under restrictive conditions such as nutrient deprivation, which occurs in the core of solid tumors. The paper by V. Banerji and S. B. Gibson deals with the emerging relationship between metabolic changes of tumor cells and autophagy, and how it contributes to increase cancer progression and chemoresistance. It has been reported that this feature depends on the increased expression of NF-E2-related factor-2 (Nrf2), a transcription factor that induces detoxifying and antioxidant systems, such as molecular chaperones and GSH-regenerating enzymes. Although Nrf2 activity is necessary for cell protection against nitroxidative stress, in several tumor histotypes, its basal activation is overinduced. Several somatic mutations have been demonstrated to destroy the interaction between Nrf2 and its physiological inhibitor, Kelch-like ECH-associated protein 1 (Keap1), thereby promoting the persistent activation of the Nrf2-mediated response and tumorigenesis. The paper by R. Brigelius-Flohé et al. discusses the dichotomous role of Nrf2 and highlights how Nrf2 activation results in the expression of several selenium-containing antioxidant enzymes (e.g., Trx reductase and GSH peroxidase) that contribute to enhance the deleterious aspects of Nrf2 in tumor progression.

Although mitochondria are the main intracellular producers of nitroxidative stress, it should be considered that they are also the primary targets of oxidative damage, a concept that has inspired the mitochondrial theory of aging. This aspect has been also taken into account in this special issue and deepened in virtue of its possible involvement in cancer and neurodegeneration. In this context, the paper of P. B. L. Pun and M. P. Murphy provides a very interesting overview on the novel role of intracellular glycation reactions as oxidative modifications specifically affecting the mitochondrial compartment, and how these can contribute to disease pathology. P. Sarti et al. discuss about the double (cytoprotective or detrimental) role of nitric oxide (NO) within mitochondria and, in particular, they describe the inhibitory effect of modifications of cytochrome *c* oxidase at different sites. The authors report a very detailed biochemical dissection of the different NO-mediated modifications of the mitochondrial complex IV and elucidate the environmental conditions in which they can occur, alongside the pathophysiological effects they can produce. This aspect is also addressed by R. M. Santos et al., who debate about the *in vivo* implications of NO signaling, focusing on the molecular mechanisms affecting bioenergetics and neurological disorders. Among these mechanisms, *S*-nitrosylation regulates protein function and modulates cellular homeostasis. In this field, Lipton's laboratory has greatly contributed to our better understanding of the implication of *S*-nitrosylation in cellular physiology. Thus, in this special issue, M. W. Akhtar provide a straightforward overview of proteins found to undergo *S*-nitrosylation that are directly involved in PD and AD pathogenesis. This paper is complemented by that of G. Di Giacomo, who specifically covered the partially neglected role of denitrosylation processes in NO-mediated signaling pathways, mainly those catalyzed by *S*-nitrosoglutathione reductase (GSNOR). They report the most recent lines of evidence arguing for this enzyme being implicated in pathophysiology and discuss how it can impact on mitochondrial function, dynamics, and selective removal by autophagy (the so-called mitophagy). Mitochondria, indeed, are highly dynamic organelles; they can fuse to form an interconnected network or undergo fission to form fragmented units that can in turn fuse again. Alternatively, fragmented mitochondria can be engulfed in autophagosome membranes for removal. These processes take place in response to different stimuli impinging on the fusion/fission machinery, mediated by a series of mitochondria-shaping proteins, such as the large GTPase family members mitofusins (Mfns) and optic atrophy 1 protein (Opa1), which underlie fusion events, and dynamin-related protein 1 (Drp1) required for mitochondrial fragmentation. The paper by M. Corrado et al. provides a detailed description of the molecular processes underlying mitochondrial dynamics. They also define how alterations in its correct occurrence can affect cellular viability and lead to pathological states, for example, cancer, neurodegeneration, and neuroinflammatory diseases. 

In addition to the continuous change of size, shape and removal by mitophagy, mitochondria are also neosynthesized. This process—mitochondrial biogenesis—is fundamental to ensure the generation of new mitochondria to maintain a constant mass of the organelle within the cell. It has been recently proposed that mitochondrial biogenesis is subjected to redox regulation as well, and this leads to hypothesize a general regulatory mechanism in which the appropriate magnitude of mitochondrial biogenesis is finely tuned by the very same early mediators of mitophagy, namely ROS and RNS. This aspect is also addressed in this special issue by the paper of E. D. Yoboue and A. Devin, who dissect out in depth the redox responsive transcription factors underlying mitochondrial biogenesis, such as the peroxisome proliferator-activated receptor gamma coactivator 1-*α* (PGC1-*α*). D. Lettieri Barbato et al. deepen this concept and extend the “redox model” of mitochondrial biogenesis towards a general responsiveness to further conditions affecting redox state, including caloric restriction. The authors suggest that oxidative stress and caloric restriction share the same signaling pathways that converge on PGC1-*α* to promote the expression of mitochondrial genes and speculate how these factors can be manipulated by means of nutritional intervention to prevent neurodegeneration.

That mitochondria are not only the “power station” of the cells is now a well-established notion, which is progressively enriched by novel insights of previously unsuspected functions and possible interplays with other cellular compartments. Overall, these elements strengthen the concept that mitochondria contribute in several aspects to the progression of pathologies. One of the most recent structural connections to be functionally characterized is the one occurring between mitochondria and endoplasmic reticulum (ER). It takes place through specific ER structures, the so-called mitochondrial-associated membrane (MAM), whose finding drove research to unravel the molecular mechanisms underlying the cellular processes bridging the two organelles. In particular, in the last years, it is becoming clear how mitochondria and ER participate to lipid-mediated signaling, calcium homeostasis, and apoptosis. In regards to this, the research paper by S. Perez-Alvarez et al. identifies in the perturbation of calcium homeostasis, the mechanism of methadone-induced neuronal cell death, which underlies its negative impact on human cognition. In particular, the authors provide evidence that methadone induces delayed calcium deregulation (DCD) by compromising the mitochondrial network and its ability to uptake calcium in a respiratory-dependent way. These results strengthen the functional association between mitochondria and ER in cell death pathways but also provide the proof of principle that additional processes (e.g., metabolism, respiration) could be regulated in concert with the two compartments. Along this line, MAM has been indicated to contain chaperons (e.g., Grp78) and proteins directly involved in oxidative protein folding, such as Ero1-*α* and protein disulfide isomerase (PDI). This is significant in the light of the fact that defects in protein folding induce the unfolded protein response (UPR), which can ultimately lead to the intrinsic (mitochondrial-mediated) pathway of apoptosis. It has been also demonstrated that, *via* MAM, Ero1-*α* competes with the mitochondrial electron tranport chain for oxygen availability and represents the main hydrogen peroxide producers within ER. Besides being crucial for protein folding, the high levels of pro-oxidant molecules within the ER are also required to reversibly regulate the function of proteins residing in this compartment. Indeed, redox regulation of ER-contained proteins and enzymes has been exhaustively reported. In this special issue, we have addressed this aspect and attempted to enlighten the involvement of the tight relationship among mitochondria, oxidative stress, and ER in disease onset. To this aim, we provide a series of papers aimed at deepening the current knowledge and reporting novel findings that shed new light on this topic. Results reported by Y. Xiong et al. show that treatment of breast cancer cells with the NO donor PABA/NO induces *S*-glutathionylation of PDI, impairs its chaperone-like activity, and induces apoptosis *via* the activation of UPR. In particular, the authors provide evidence that the activation of cell death is associated with inability of the *S*-glutathionylated PDI to bind to the estrogen receptor *α* (ER*α*) with consequent inhibition of the expression of genes involved in cell proliferation. Nevertheless, if the impairment of PDI activity and the aberrant activation of UPR could be exploited for cancer cell killing, it has been copiously reported to be detrimental for neuronal viability. Indeed, the accumulation of unfolded protein and excessive UPR are among the causes triggering neuronal cell death in a large number of neurodegenerative diseases. In this context, E. Ferreiro et al. elucidate the association between pathological changes found in AD brain (e.g., protein inclusion) and mitochondrial dysfunction, and how oxidative stress resulting from this condition is intimately linked to ER-derived ROS production and UPR. The paper by F. Di Sano and M. Piacentini adds new insights to this aspect by focusing on the role of reticulons family, and of RTN-1C in particular, in different neuron pathologies. RTN-1C is localized on ER membranes and regulates ER structure and function; in their paper, the authors provide strong lines of evidence that this protein might be a promising target in the treatment of different pathologies.

In conclusion, the field of redox biology has grown beyond all expectations and now interests virtually every biological process. Perturbation in redox balance and signaling has indisputable relevance in the pathobiology of cancer and neurodegenerative diseases, and restoration of homeostasis constitutes an appealing therapeutic strategy. This special issue recapitulates the state of the art of redox biology in relation to these disorders and highlights the most critical priorities to achieve better understanding of pathogenesis and develop successful therapies. We are therefore confident that this collection will be highly informative for those in the field as well as for the broad scientific community.

*Giuseppe Filomeni* *Giuseppe Filomeni* *Juan P. Bolaños* *Juan P. Bolaños* *Pier Giorgio Mastroberardino* *Pier Giorgio Mastroberardino*

